# Intracholecystic Papillary Neoplasm of the Gallbladder Neck With High‐Grade Dysplasia: A Diagnostic and Surgical Challenge

**DOI:** 10.1002/ccr3.72692

**Published:** 2026-05-08

**Authors:** Pradeep Yadav, Sajan Yadav, Kshitiz Parajuli, Aron Neupane, Amit Kumar Sah, Neha Yadav

**Affiliations:** ^1^ Department of Surgery Birat Medical College Teaching Hospital Morang Nepal; ^2^ Birat Medical College Teaching Hospital Morang Nepal; ^3^ Nepal Medical College and Teaching Hospital Kathmandu Nepal

**Keywords:** abdominal bloating, cholecystectomy, intracholecystic papillary tubular neoplasm

## Abstract

Intracholecystic Papillary Neoplasia (ICPN) is a pre‐invasive gallbladder lesion (< 0.5% of cholecystectomies). A 70‐year‐old female presented with complaints of abdominal bloating and vomiting. Imaging revealed cholelithiasis with gallbladder neck mass. Diagnostic laparoscopy with extended cholecystectomy and lymphadenectomy was performed. Histopathology revealed ICPN—biliary type with high‐grade dysplasia. The 5‐day follow‐up was uneventful.

## Key Clinical Message

Intracholecystic Papillary Neoplasm (ICPN) is a rare (< 0.5%) pre‐invasive lesion that can radiologically mimic gallbladder cancer. This case of a 70‐year‐old female underscores the necessity of high clinical suspicion and oncologic resection to achieve R0 margins. Early surgical intervention offers an excellent prognosis.

## 1 Introduction

Intracholecystic Papillary Neoplasm (ICPN) is a neoplastic lesion defined as pre‐invasive and usually presents as a papillary growth within the gallbladder. The neoplasia, identified as a lesion within Intraductal Papillary Neoplasm of Bile Duct (IPNB), parallels the Intraductal Papillary Mucinous Neoplasm (IPMN) of the pancreas and is now termed Intraductal Papillary Neoplasms by the World Health Organization in 2010 and revised again in 2019 under a single classification of precursor lesions to the carcinogenesis of pancreato‐biliary tract [1, 2]. These tumors comprise pre‐invasive neoplastic/dysplastic cells that are clinically detectable (≥ 1.0 cm) masses [3].

ICPN is a lesion similar to IPNB which shows characteristic dilatation of extrahepatic and/or intrahepatic bile due to a papillary biliary neoplasm covering thin fibrovascular stalk [4]. ICPN accounts for < 0.5% of all cholecystectomies and is commonly occurs in body or fundus of the gallbladder and neck lesions are rarer [5].

We report a rare case of ICPN with a papillary lesion in the neck of the gallbladder in a 70‐year‐old female with atypical presenting symptoms. Our case report has been written in line with CARE guidelines [6].

## 2 Case Presentation

### 2.1 Case History/Examination

A 70‐year‐old female, from the rural district of Nepal, presented to the out‐patient clinic with complaints of abdominal bloating for 2 weeks which was sudden in onset, gradually progressive in nature associated with nausea, and decreased appetite. She did not report abdominal pain and vomiting, and there was no history of diarrhea, constipation, melena, cough, shortness of breath, or chest pain. She has been diagnosed hypertensive for over 10 years and is under medication with amlodipine 5 mg and losartan 50 mg. She is non‐alcoholic and a non‐smoker and has no history of surgeries in the past.

On examination she was alert, conscious and well oriented to time, place and person. She had a blood pressure of 110/70 mmHg, pulse of 82 beats per minute, respiratory rate 22/min and was afebrile to touch. Chest examination revealed a normal chest with bilateral vesicular breath sounds and equal air entry. The CNS examination was intact.

On abdominal examination, gallbladder was palpable, non‐tender, and bowel sounds were present.

Blood biochemistry revealed an elevated alkaline phosphatase of 252 units/I; CA‐19.9 was within normal range measuring 4.56 U/ML. Coagulation studies demonstrated a normal PT/INR.

## 3 Methods (Differential Diagnosis, Investigations, and Treatment)

Contrast Enhanced Computed Tomography (CECT) of Abdomen showed a distended gallbladder with minimally enhancing heterogeneous mass in the gallbladder fossa region abutting up to the anti‐pyloric region. The suspicion of a locally advanced carcinoma was a clinical and radiological impression formed by the multidisciplinary team during the acute management phase. Multiple cholelithiasis was also revealed and no adjacent liver infiltration or lymphadenopathies were marked. Magnetic Resonance Cholangiopancreatography (MRCP) confirmed cholelithiasis with GB neck mass with involvement of cystic duct causing distension of the body and fundus.

**FIGURE 1:**
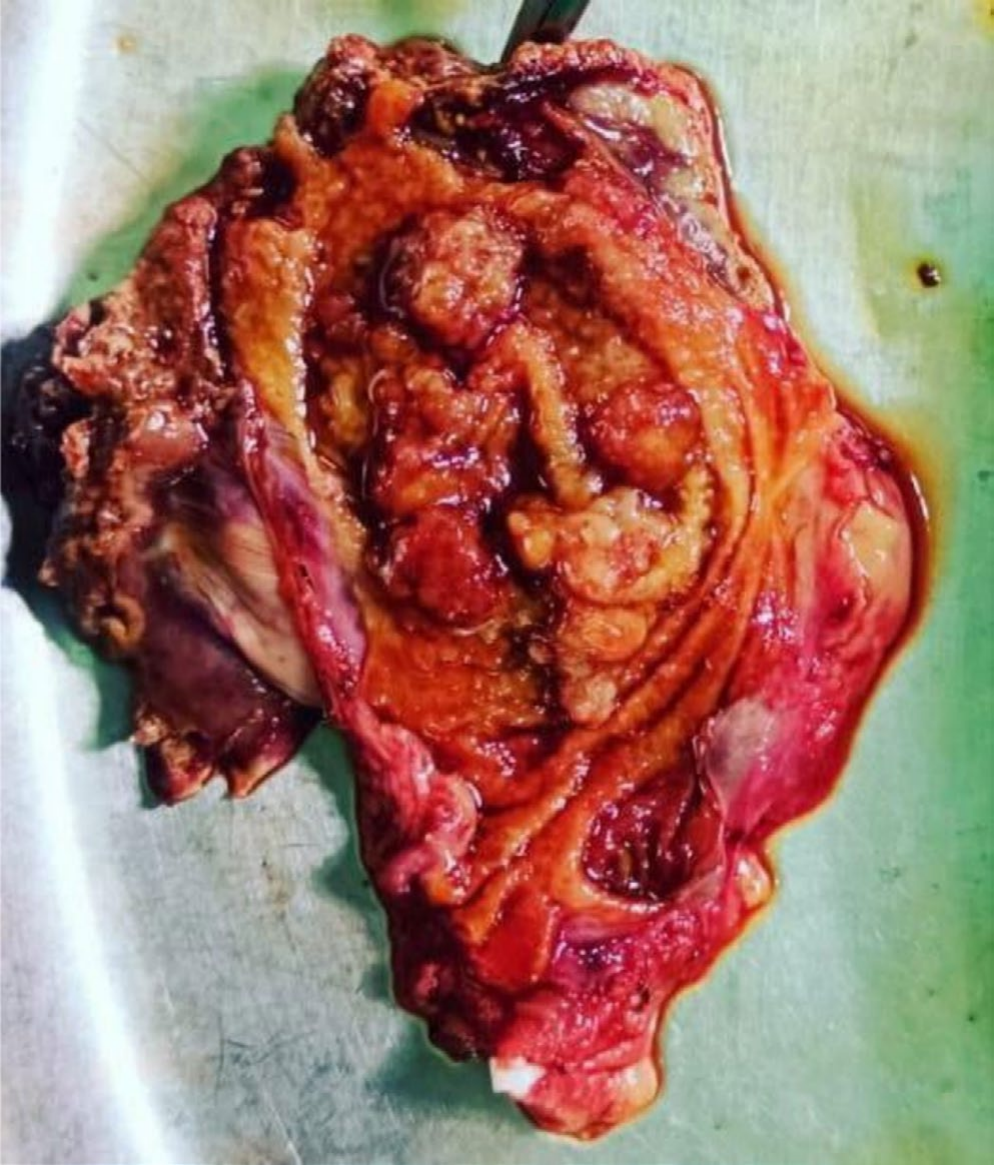
Gross specimen showing intraluminal growth in GB lumen.

**FIGURE 2:**
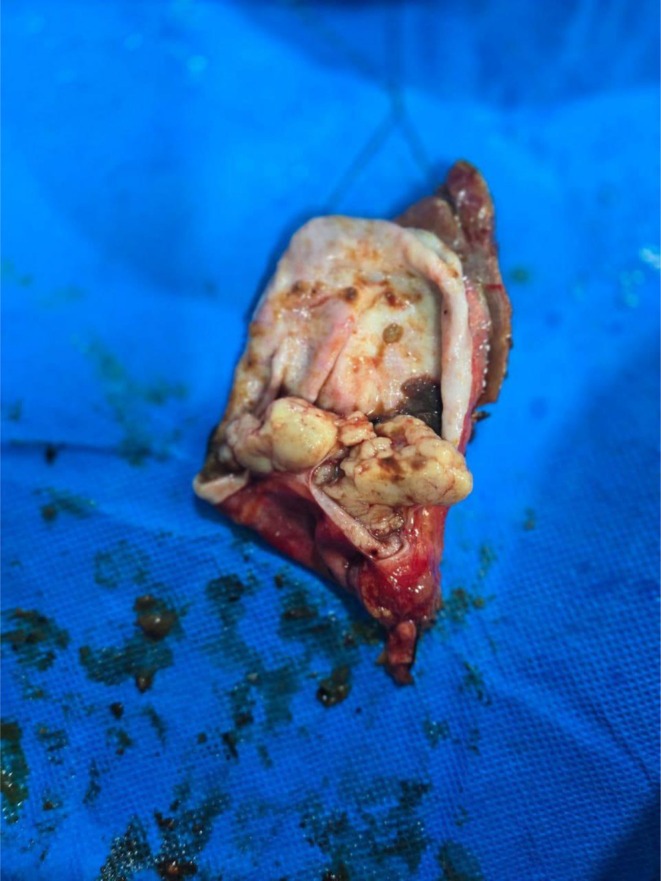
Gross specimen showing whitish growth with fatty infiltration.

Initially, locally advanced carcinoma of gallbladder neck was suspected and the patient was planned for a diagnostic laparoscopy followed by extended cholecystectomy and lymphadenectomy. At the operating room, a distended gallbladder was noted with a hard irregular polypoidal mass measuring 3.2 cm × 3.1 cm in the neck of gallbladder and was not seen involving serosa towards peritoneum. Multiple calculi were found in the gallbladder lumen (Figures 1 and 2). Common bile duct, liver and the hepato‐duodenal ligament were free of tumor. No ascites was noticed. An extended cholecystectomy and lymphadenectomy were performed as planned. A wedge resection of 2 cm of liver was performed which involved liver segment IVb and V. The cystic duct margin measuring 0.8 × 0.5 × 0.4 cm was sent for a frozen section procedure which revealed cystic duct margins free of tumor. The resected gallbladder with lymph node with wedge resection of liver was sent for histopathological examination.

## 4 Results and Conclusion (Outcome and Follow‐Up)

Patient was shifted to post‐operative surgical ICU where she was managed with IV antibiotics, analgesics, IV fluids and daily drain output monitoring. On the second post‐operative day, she was transferred to the post operative ward. Post‐operative stay was uneventful and she was discharged on the 8th day of operation and a follow‐up after 5 days was scheduled.

**FIGURE 3:**
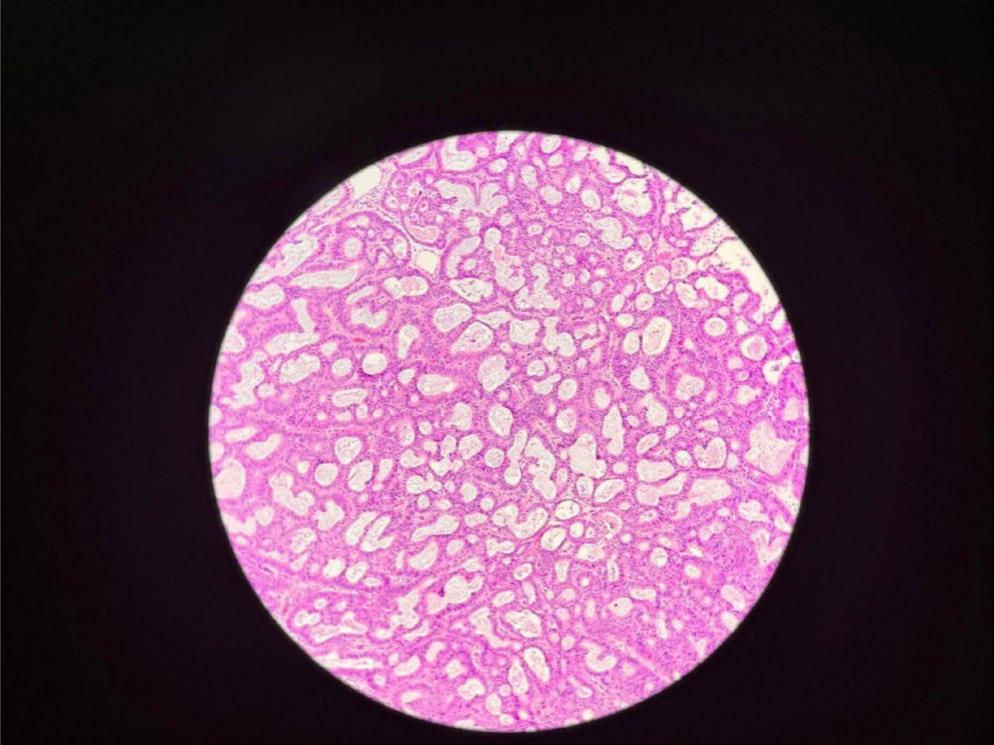
Histopathological examination specimen showing special cells with tubular and papillary pattern of growth.

Her histopathology report revealed intracholecystic papillary neoplasm of gallbladder (ICPN), biliary type with high‐grade dysplasia (Figure 3). Cystic duct margins and hepatic resection margins were free of tumor. 17 isolated lymph nodes were free of tumor.

The 5‐day follow‐up was uneventful.

## 5 Discussion

ICPN was described the very first time by WHO in 2010 as a pre‐invasive neoplasia in Gallbladder, a lesion of IPNB. These adenomas and intracystic papillary neoplasms with dysplasia are pre‐malignant lesions that show an exophytic growth pattern [1]. These tumoral intraepithelial neoplasms are analogous to IPMNs and ITPNs of the pancreas as well as IBPNs [3]. These tumoral neoplasms are identical in terms of their exophytic nature, pre‐invasive course, the presence of a spectrum of dysplastic changes, and expression of various cell lineages occurring to a varying extent. The varied cell lineages may be biliary, gastric, or oncocytic. These ICPNs are intracystic pre‐invasive lesions showing exophytic growth of ≥ 1.0 cm. A growth of ≥ 1.0 is used as a criterion due to its wide acceptance by surgeons and radiologists to determine the indication of cholecystectomy and WHO's consideration in its 2010th year iteration of classification of tumors of the digestive system [1, 3].

The term ICPN correlates to its ampullary‐pancreato‐biliary counterparts. The nomenclature was followed after suggestions of Dr. Juan Rosai (Personal communication, Washington, DC, March 2010), who used the term “Intracholecystic” to denote the anatomical localization of the lesion which involves the GB mucosa. “Papillary‐ tubular” in its name highlights the exophytic pre‐invasive nature of the tumor along with the possibility of both papillary or tubular configuration or both. “Mucosal” was not considered as a part because of only minor production of mucin from the lesion which is also less common in the biliary IPNs in contrast to IPMNs which are known for copious mucin production [3].

The incidence of ICPN is very rare and accounts for < 0.5% of all cholecystectomies [5]. Majority of invasive carcinomas in the ampullary‐pancreato‐biliary tract arises from these types of intraepithelial neoplasia unlike in the tubular GI tract in which carcinoma follows a more common adenoma‐carcinoma sequence. The mean age of patients with ICPN is 66.3 years old according to a study by Kumagai et al., which further presents female patients outnumbering males [7]. In this report our patient is a 70‐year‐old female which also supports the study's conclusion as it surpasses the mean age of diagnosis and a female occurrence in this first of a kind diagnosis in our centre.

Patients with ICPN often present with right upper quadrant pain and demonstrable palpable gallbladder on abdominal palpation. In a few cases, patients may present with obstructive jaundice in conditions where tumors extensively invade the bile duct. These protruding or advanced tumors cause obstruction of biliary passages, thus resulting in obstructive jaundice [7, 8]. In our case, the female presents atypically with only abdominal bloating, which was sudden onset, gradually progressive, and marked by nausea and vomiting. Our patient, unlike other literature, did not report pain in the abdomen. She did present with a palpable gall bladder on abdominal examination.

Compared to conventional imaging modalities like CECT and USG, Endoscopic Ultrasound (EUS) and Peroral Cholangioscopy (POCS) easily show papillary tumors, providing a much better characterization of ICPN. Other techniques like Intraductal Ultrasound (IDUS), Endoscopic Retrograde Cholangiopancreatography (ERCP), Fluorodeoxyglucose Positron Emission Tomography (FDG‐PET), and Magnetic Resonance Cholangiopancreatography (MRCP) can also be used as a diagnostic tool for tumor visualization [7]. We performed CECT abdomen initially and MRCP to visualize the mass. However, histopathology provided the diagnosis in our case.

The mainstay for treatment of ICPN remains oncologic resection of the mass. Selection of optimal surgical procedure is very challenging and is often based on the presentation of ICPN. A simple cholecystectomy is sufficient for ICPN limited to GB mucosa. Patients suspected of bile duct invasion may undergo pylorus preserving Pancreatico‐Ducodenectomy (PPPD). A patient with suspicion of Gallbladder carcinoma often undergoes extended cholecystectomy in addition to PPPD [5, 7]. In our case, we considered diagnostic laparoscopy followed by extended cholecystectomy and lymphadenectomy.

Invasive carcinoma is seen in more than half of the ICPNs at the time of diagnosis. Most of these are pancreato‐biliary type GB adenocarcinomas. Mucinous and neuroendocrine types may also be found in a few cases. ICPN presents a spectrum of cytoarchitectural atypia in histopathology along with varying degrees of dysplastic changes which are associated with either papillary or tubular growths or both. More than three quarters of these cases are predominantly papillary. The frequency and amount of high‐grade dysplasia (HGD) and associated invasive carcinoma are significantly higher in papillary and tubule‐papillary cases than in tubular cases [3, 7, 9]. Unlike pancreatic counterparts, a mixture of cell lineages is seen in coexistence in significant proportion in case of ICPN which is reflected by heterogeneous staining pattern by immunohistochemistry (IHC).

Most common cell type remains the biliary type which is commonly expressed MUC1 [3]. ICPN that has higher associated risk of invasion includes greater amount of papillary configuration, larger extent of HGD, and a predominance of non‐pyloric cell lineage [3]. In our case, the histopathology report revealed ICPN of biliary type with high‐grade dysplasia. Cystic duct margins and hepatic resection margins were free of ICPN. 17 Lymph nodes were isolated and were free of tumor. Prognosis of ICPN is comparably better than that of any other invasive carcinomas of the gallbladder. Non‐invasive types of ICPN have a much better prognosis than invasive ones [3].

## 6 Conclusion

The presenting symptoms of such a condition differ from patient to patient and can mimic gallbladder cancer both symptomatically and radiologically. Nevertheless, it has a better prognosis than the invasive ones. Thus, the early detection of the condition is very much crucial.

This case highlights the importance of intraoperative judgment in gallbladder lesions in the conditions of diagnostic uncertainty. This case reinforces best practices in surgical oncology: ensuring R0 resection (no residual tumor) even for pre‐malignant lesions to prevent recurrence. Thus, this case report shows how clinical suspicion, surgical technique, and pathology correlation collectively guide optimal patient management.

## Author Contributions

**Pradeep Yadav:** conceptualization, writing – original draft. **Sajan Yadav:** writing – original draft. **Kshitiz Parajuli:** writing – original draft. **Aron Neupane:** writing – review and editing. **Amit Kumar Sah:** writing – review and editing. **Neha Yadav:** writing – review and editing.

## Funding

The authors have nothing to report.

## Consent

Written informed consent was obtained from the patient to publish this report in accordance with the journal's patient consent policy.

## Conflicts of Interest

The authors declare no conflicts of interest.

## Data Availability

Data will be provided by the corresponding author upon reasonable request. Images uploaded in the separate files.
